# The role of social support among caregivers of people with cancer from Chinese and Arabic communities: a qualitative study

**DOI:** 10.1007/s00520-024-08502-6

**Published:** 2024-04-26

**Authors:** Eva Y. N. Yuen, Megan Hale, Carlene Wilson

**Affiliations:** 1 School of Nursing and Midwifery, Faculty of Health, Deakin University, Melbourne, Australia; 2 Centre for Quality and Patient Safety, Monash Health, Melbourne, Australia; 3 School of Psychology and Public Health, La Trobe University, Bundoora, VIC Australia; 4 Psycho-Oncology Research Unit, Olivia Newton John Centre, Austin Health, Heidelberg, VIC Australia; 5 Centre for Epidemiology and Biostatistics, University of Melbourne, Parkville, VIC Australia

**Keywords:** Cancer, Caregivers, Culturally and linguistically diverse, Chinese, Arabic, Qualitative, Social support, Emotional support

## Abstract

**Purpose:**

Cancer caregivers from culturally and linguistically diverse (CALD) communities have reported significant unmet emotional support needs. This study aimed explore the role of social support to manage emotional wellbeing among cancer caregivers from Arabic and Chinese communities in Australia.

**Methods:**

Semi-structured interviews were conducted with Chinese (*n* = 12) and Arabic (*n* = 12) speaking cancer caregivers. Participants’ mean age was 40.6 years; majority were female (83%) and providing care to a parent (41.67%).

**Results:**

Using thematic analysis to analyse interview data, five overarching themes emerged describing caregivers’ perspectives on social support. Themes were related to the following: (1) receiving emotional support from social networks, (2) barriers to accessing emotional support from social networks, (3) isolation and loss of connection following the cancer diagnosis, (4) faith as a source of support, and (5) utility of support groups and caregiver advocates. Several caregivers relied on social networks for emotional support; however, caregivers identified key cultural and generational barriers to seeking support from their social networks which prevented caregivers from disclosing their emotions and caregiving situation. Caregivers also reported being isolated from their support system.

**Conclusion:**

Empirical testing of culturally appropriate strategies that improve social support seeking among caregivers from CALD communities is recommended.

Cancer is a major cause of morbidity and mortality in Australia accounting for 19% of the population’s illness and 9% of the health system’s expenditure [1]. People diagnosed with cancer often rely on family members and friends to provide informal medical, practical, psychosocial and informational support when managing diagnosis and treatment [2–5].

For these informal caregivers, the significant negative emotional impact of providing care has been highlighted. Caregivers often experience physical and emotional exhaustion, feelings of ill-preparedness, and persistent levels of distress that are higher than the general population [6, 7]. Despite this, there exists several barriers to caregivers accessing their own support. First, healthcare systems largely perceive the needs of caregivers as secondary to patient wellbeing [8]. In addition, caregivers themselves may neglect their own health and wellbeing in order to prioritise the needs of the care recipient [9, 10]. Finally, caregivers may not know where to find relevant support. In a recent Australian study, caregivers reported lack of knowledge about where to find emotional support to meet their own needs [11].

Nonetheless, research shows that outside of formal health services, social support plays an important role in assisting caregivers. Social support has been described as “the existence or availability of people on whom we can rely, people who let us know that they care about, value and love us” [12] (pp 127). Gottlieb and Bergen [13] described a range of different types of social support including; instrumental (e.g. transport assistance), appraisal (affirming one’s sense of self), informational (e.g. advice to solve a problem), emotional (e.g. care, love, empathy) as well as companionship. Among cancer caregivers, social support and connection with others have been shown to alleviate burden, improve psychological outcomes [14], improve coping skills [15, 16], reduce unmet needs [17], and enhance quality of life in caregiver populations [18–20].

Australia is a culturally and linguistically diverse (CALD) nation, with approximately 30% of the population born overseas, and one-fifth speaking language other than English at home [21]. People from CALD communities are defined as those whose main language spoken at home is not English and/or who were born in non-English speaking countries [22]. Research has shown that people from CALD communities with cancer have higher unmet supportive care needs, greater emotional support needs, poorer quality of life, and more communication barriers than their English-speaking counterparts [23–25]. Fewer studies have examined the supportive care needs of caregivers of people with cancer from CALD communities. In a recent qualitative study with Chinese caregivers and patients living in Australia, caregivers reported difficulty identifying where to seek support for their emotional distress [26]. Further, social support has been identified as a contributing factor in the development of resilience and enhanced quality of life in people with breast cancer from Chinese communities [27]. However, few studies have examined how caregivers from CALD communities’ access and use their social support networks to manage their own health and wellbeing.

Despite research showing that caregivers of people with cancer from CALD communities may experience reduced quality of life and psychosocial outcomes, there is limited data concerning how these caregivers can be better supported. Nonetheless, the limited available research suggests that caregivers from CALD communities experience additional needs due to language difficulties and differences in communicative and cultural values [11, 28], which may impact their capacity to find, access and utilise critical social support. Thus, the current study aims to explore the role of social support to promote wellbeing in caregivers of people with cancer from Arabic and Chinese communities residing in Australia. Specifically, the study sought to examine how and whether caregivers from CALD communities engage with their social support networks for emotional support when providing care to someone with cancer. Arabic and Chinese speaking populations were targeted since they are two of the largest CALD language groups in Australia (Arabic, 1.4% population; Cantonese/Mandarin, 3.7%) [29].

## Methods

The current study used a qualitative design comprised of semi-structured interviews. Participants were 24 caregivers who were currently providing, or had provided, unpaid care to someone diagnosed with cancer who spoke Chinese or Arabic as a first language. Eligibility criteria included individuals who provided care to someone in the preceding three years, were over the age of 18, were currently residing in Australia, and were able to participate in an English language interview without an interpreter. Ethics approval was obtained from the La Trobe Human Research Ethics Committee (HEC20465). The study aimed to recruit 12 participants for each cultural group. This target number was based on prior research findings concluding that for homogeneous studies using purposeful sampling, 12 interviews were sufficient for data saturation [30]. Participants received a $50 Coles eGift voucher as a token of thanks for their time.

## Data collection

Recruitment was undertaken by LOTE, a multicultural marketing agency in Australia. Participants were recruited through ethnic media channels, multicultural organisations, aged care centres, local councils, and ambassadors of CALD communities. Interested individuals were invited to contact LOTE researchers to express their interest in participating in the study, who were then screened for eligibility. Eligible participants were emailed a participant information and consent form by LOTE. Individuals who returned a signed consent form to LOTE were then contacted by a researcher (MH) to arrange a video interview (Zoom). Verbal consent was obtained at the start of each interview. Interviews followed a semi-structured interview process, and participants were asked about the type of emotional and social support they received from their networks. Interviews were conducted between March and May 2021 and audio recorded.

### Data analysis

Interviews were transcribed using Otter.ai. Identifying details were removed from all transcriptions, and all recordings were removed from Otter.ai once transcription was completed to maintain participant privacy. Data were analysed using Braun and Clarke’s [31] six-phase process of thematic analysis commencing with two researchers (MH, EY) reading and re-reading the transcripts to become familiarised with the data. Data were then coded in a systematic manner for the entire dataset. Codes were then collated into potential themes, with data checked to ensure their relevance in themes. Proposed themes and associated evidence were reviewed, refined and defined by the research team, with resultant themes confirmed through discussion. An iterative, reflexive and immersive process was used to make meaning and gain insight into participant experiences. To maintain reflexivity, the researchers sought to position themselves as a learner, with the participants as the teacher, whilst maintaining the view the research was being conducted with, and not for participants [32].

## Results

Table 1 displays participant demographic information in order of interview (also presented in Yuen et al., accepted for publication [33]). Participants were aged between 29 and 65 years, with a mean age of 40.6 years (three participants did not specify their age). Of the sample, 12 (50%) were of Arabic background, and 12 (50%) were of Chinese background. Most participants (83%) were female. The majority were providing care to a parent (41.67%), with interview length ranging between 25 and 75 min.

**Table 1:** Participant demographics

Caregiver	Cultural group	Age	Gender	Care recipient was my:	Care recipient cancer type
1	Chinese	30	Male	Mother	Kidney
2	Chinese	29	Female	Mother	*
3	Chinese	35	Male	Grandfather	Stomach
4	Arabic	35	Female	Husband	Colon
5	Chinese	30	Female	Stepfather	Brain
6	Chinese	29	Female	Sister	Cervical
7	Chinese	*	Female	Aunt	Stomach
8	Arabic	35	Female	Husband	Thyroid
9	Chinese	*	Female	Cousin	Prostate
10	Chinese	28	Female	Mother	Head and neck
11	Chinese	44	Male	Father	Gastric
12	Arabic	51	Female	Daughter	Hodgkin's lymphoma
13	Arabic	*	Female	Mother	Bladder
14	Chinese	28	Female	Friend	Cervical
15	Chinese	31	Female	Mother	Leukaemia
16	Chinese	29	Male	Grandmother	Pancreatic
17	Arabic	54	Female	Daughter	Thyroid
18	Arabic	65	Female	Daughter	Non-Hodgkin’s Lymphoma
19	Arabic	55	Female	Mother-in-law	Leukaemia
20	Arabic	24	Female	Mother	Breast
21	Arabic	55	Female	Mother-in-law	Pancreatic
22	Arabic	56	Female	Sister	Ovarian
23	Arabic	49	Male	Father	Kidney
24	Arabic	62	Male	Wife	Breast

^*^Unclear/not provided by participant

Five overarching themes that described caregivers’ perspectives on informal support emerged from the thematic analysis. Themes were related to the following: (1) receiving emotional support from social networks, (2) barriers to accessing emotional support from social networks, (3) talking about the cancer, (3) isolation and loss of connection following the cancer diagnosis, (4) faith as a source of support, and (5) utility of support groups and caregiver advocates.

### Receiving emotional support from social networks

Across participant interviews, three subthemes emerged within the theme of receiving emotional support from social networks: (a) utility of engaging with others to cope, (b) support from family members, and (c) support from friendship networks. These subthemes are described below.

#### Utility of engaging with others to cope

Some caregivers identified the usefulness of talking about the caregiving situation to better cope with their circumstances (see Table 2 for supporting quotes). Sharing their circumstances helped caregivers to better manage the emotions associated with the caregiving experience. Caregivers also spoke of encouragement and support received from others who had experienced a family member/friend with a diagnosis through sharing experiences.

**Table 2:** Quotes to support themes and subthemes

Theme/sub-theme	Quote/s
Receiving emotional support from social networks	
Utility of engaging with others	“I think sometimes talking, you get it off your shoulders, if you talk. Even if nothing change (sic) in a situation but talking with someone I believe it helps. It helps you get it out of your system and it’s…off your shoulder like…you share it with someone. And it’s didn’t go, but it’s feels lighter than before” (C19, Arabic, female, caring for mother-in-law) “…we should talk to our friends…some friends with their grandkids got cancer as well. So we can interchange the experience. We can encourage each other especially in the hospital…” (C11, Chinese, male, caring for father)
Support from family members	“My husband, my husband, he’s like, his perfect man. Yeah, yeah, he’s very, he’s very good…and he supports me all the time” (C13, Arabic, Female, caring for mother) “…and my sister when I’m like down, I talk to her and she helps me like, she lift my, lift my spirits up a little bit to keep going (C2, Chinese, female, caring for mother) “Yeah, because they sometimes will also give advice on how to helping [our relatives cope with the diagnosis]. And, yeah, I think that helps” (C3, Chinese, Male, caring for grandfather) “…they all knows about the information, but they couldn’t really help because the other part of our family are still in China. So yeah, basically are just to online video chat, or just like, yeah, emotional support” (C7, Chinese, female, caring for aunt)
Support from friends	“…one of them, her husband was diagnosed with lymphoma, like three or four years ago, so she knew she knew, kind of knew how that treatment was going…So they were checking on me every now and then and checking on her” (C2, Chinese, female, caring for mother) “…both of their parents, both of their mum has had a cancer before. And yeah, they just gave me some emotional support. And they also gave me a link…[to]a proper cancer website from United States…[with a guide on what treatments should, should you do” (C10, Chinese, female, caring for mother) “…because all my friends know my mom. Then they’ll come and visit my mom. They’ll come and trying to help, they will always be offering what…can we do?” (C15, Chinese, female, caring for mother) “I’m comfortable talking about it to anyone. But um, I just don’t think that’s helpful, for me” (C6, Chinese, female, caring for sister)
Barriers to accessing support from social networks	
Responsibility to protect others from burden	“I don’t want to give them any burden…I always pretend that I’m happy, everything I can cope with…I just don’t want my family member worry about me, otherwise, there are many people worried” (C9, Chinese, female caring for cousin) “So, I can’t cry in front of my kids, or I feel sad. I can’t say anything, just be happy. Smile, take it easy…but in my head it is like constant worry…I have to be all the time optimistic I have to be happy, that is very important” (C4, Arabic, female, caring for husband) “…I can’t tell my wife or tell my parents, the parents is worrying enough, I don’t want to bother them. For my wife, I hope she could enjoy her life…So I don’t want my negative feelings…my negative things to bother her as well…this is the first time I talk about this” (C1, Chinese, male, caring for mother) “Even if I tried to mention it to someone and they start to feel sad and that’s it made me very sad. So that’s why I tried to…keep a distance” (C2, Chinese, female, caring for mother) “Yeah, tradition in a lot of Chinese…we just protected our self. My parents protect me very well they don't give me any negative [news]…not negative but they don’t want to share that news to me, so they always said everything’s good…But I know that it’s not true, but they are reluctant to talk about that” (C9, Chinese, female, caring for cousin) “But even in my family because the old generation to the young ones they don’t want make the big pressure on us” (C3, Chinese, Male, caring for grandfather) “But most of them tend to avoid to do so, because that family tradition…and if I face less big things I don’t know how to do I just worry and panic. So, because I don’t have experience” (C2, Chinese, female, caring for mother) “This is the first, first ever interview ever to you know, [that I have spoken about my experiences] as a carer… …it’s quite interesting because I don't get to share that…Good to talk to someone about those experience. (C14, Chinese, female, caring for friend) “It’s the first time I speak about it actually…now I’m looking at back in the days…It’s quite helpful for me to speak about it (C8, Arabic, female, caring for sister)
Reliance on oneself, stoicism and familial duty	“I just feel like if you’re not satisfied with your life, or work, or you’re experiencing a very hard situation, other people can’t really help. It’s yourself, you need to go through…maybe it’s just, maybe my past experiences…[Although] they are really helpful. After they talk to me, I feel I feel better, I feel I don’t need to be in a rush to go back to China to quit the job. And maybe my mum will be better” (C10, Chinese female, caring for mother) “I like being good at stuff on my own, I don’t like being told to…or being the centre of the attention, you know” (C8, Arabic, female, caring for sister) “I don’t want my wife and my wife’s family to get involved. This is not their business, in my opinion. I don’t want my mother’s issue to bother my wife’s family…it’s okay…I’m a tough person I believe” (C1, Chinese male, caring for mother) It um, it felt like it’s all my responsibility for me, because I am her family and you know, we’re bonded by blood and I feel very guilty for all the friends that are helping, ah grateful, but also guilty as it shouldn’t be them, it should be me (C6, Chinese female, caring for sister)
Avoiding discussions as a coping mechanism	“No, I didn’t [talk to], even my friends. I’m really close to them, but I didn’t talk because I felt like I feel weak when you talk and you just want to be strong…Yeah, I didn’t talk to any anyone about it” (C20, Arabic, female, caring for mother) “…then I’m like maybe I'm that person that doesn’t talk about a lot about my stress…like…at home I’m a bit stressed…so but like I don’t talk about it a lot” (C22, Arabic, female, caring for mother-in-law) Because we had friends but like, it just depends on the person like for me, I don’t like to do talk about stuff with other people. Yeah, I kind of bottle up everything. Which is not great, yeah (C18, Arabic, female, caring for daughter-in-law)
Cancer, death and illness as taboo topics	“…we tend to avoid this conversation. It’s like, it’s like a bad fortune. If you talk about it, it will come back. No, no, no, no, no, it’s just some stupid things. For example, my mom says, ‘ah you can’t talk about this or it will come back’…It’s not a taboo…It’s not a topic that we avoid, but we just don’t talk about it a lot” (C1, Chinese, male, caring for mother) “My Grandpa passed away and my Dad only tell me…Grandpa’s condition is not very well just to [fly back to China]. And once I just landed the airplane, they just want me [to] go to sleeping and then tomorrow we’ll go to see your Grandpa. And the next day my parents told me my Grandpa passed away…So that is like, my my family's traditions, they don’t want to talk about death. They don’t want to worry me… “And I’m also have fear, because I’m not the person who can take big responsibility…I always avoided to thinking about death or thinking about difficulties…and I know sometimes I have to face it…so like my cousin, but I just avoid” (C9, Chinese, female, caring for cousin [brother in Chinese culture]) “…I think she’s very conservative, or maybe that it’s a (sic) effect of our family. For example, I know her brother had a problem liver…but he never mentioned that…So I told my wife, I feel that something’s wrong, with your brother…She said yes, he has a problem, he has liver transplantation” (C24, Arabic, male, caring for wife)
*Isolation and Loss of Connection following the cancer diagnosis*	
	“It made me sad, very sad at the beginning. Because I…don’t have a big family here” (C12, Arabic, female, caring for daughter) “Because I feel like, all the time lonely…maybe if I have my family, or family member, it would be better” (C4, Arabic, female, caring for husband) “[My wife] asked me to keep [the diagnosis a] secret between us…maybe lost friends or close friends. Because we have sometimes we’re going to the hospital…my sister, they doesn’t know. My mom doesn’t know, till now. She will she asked me not to tell anyone…So I can’t go to any one to discuss this issue…” (C24, Arabic, male, caring for wife) “I just feel lonely…before I…always rely on my cousin…he is the person…to do everything for me. But after he got this disease, I have everything I need to do by myself” (C9, Chinese, female, caring for cousin) “…I can’t, like talk everything in my head to him. I said, like, maybe [the cancer will] be worse or…or maybe it’s come back again.…because the doctor said we don’t know whether it will come back again or not. So in my head I said, what’s happen if it comes back again” (C4, Arabic, female, caring for husband)
Faith as a source of emotional support	
	“[Inaudible], thank God for that, and my prayer. I pray. I used to go to church a lot and pray a lot. And that’s it. That’s it. Look when when this happened, you don’t have much time to think about yourself” (C17, Arabic, female, caring for daughter) *Interviewer:* “Did you have anyone supporting you like if if you were scared or feeling worried about [your wife]? Where did you go to for help? *Caregiver:* To God. To myself and to God…To me, as a Christian, Christian, that faith is really important to me. So I put everything in hands of God” (C24, Arabic, male, caring for wife) “…when you have faith.…it kind of helped…you pray, and you hope for the best…religion is important to us…The priest of the church, he came to visit her in the hospital…We said to him, like, as if he’s a messenger from God, I said to him, why, why is this happening to us? Like, we didn’t want anybody to be sick or, so, and he said, that’s just like a test and you will be over it, and it’s just like, make you strong person, of course, my daughter wasn’t convinced, like she said no, no, no. I said, No, it is, it is. So you will not see it, now” (C12, Arabic female, caring for daughter)
Utility of support groups and cancer advocates	
	“I was just reading the comments or what people are going through, or what do they feel?…They answer each other…Also…there was an informal nurse on that group…when there are some questions that require…more information or more advice, she would pop in and like comment on it. So it’s helpful in a way” (C12, Arabic, female, caring for daughter) “…Maybe due to the culture or language ah difference…we didn’t join any group like that…I don’t think Chinese people would seek help from strangers. Normally, friends, maybe family members, or your neighbours could be possible” (C7, Chinese, female, caring for aunt) “…I don’t think that will be helpful, everybody’s situation is different. I don’t think…other caregivers, only a few caregivers could do what I did. Most of them just provide what they can provide on the surface…It’s not likely for them to provide some encouraging news…They can do it, doesn’t mean that we can do it too” (C1, Chinese, male, caring for mother) “So that’s what I said, I don’t have time for it. I think about [the cancer] a lot and [if I] make (sic) a group, maybe I will be worse off” (C4, Arabic, female, caring for husband) “I’ve never been to the kind of support group. I saw other patients…there’s a lot of different families, some, some will just…like crying in the ward…some will just cry in the hallway. I don’t want to deal with that. So I just assumed that will be like a big pile group of people like cuddling, and then crying, hell no” (C6, Chinese, female, caring for sister) “I don’t have support…my English is just so-so, so I cannot talk with the local people quite to show…what I’m…really thinking…if they can find the same group of people with the same background, share the experience” (C9, Chinese, female, caring for cousin) “Maybe if there was, like, in the community, someone from the same background has been through this experience before, so they can give you their advice” (C12, Arabic, female, caring for daughter) “I think if you’re from the same background, those speak the same language…it will definitely make difference” (C14, Chinese, female, caring for friend)

#### Support from family members

A number of caregivers identified turning to their family members for emotional support as a source of comfort and as a means to help them through the challenges of providing care to someone with cancer (Table 2). Caregivers also sought emotional support by speaking with family members to help support them when they felt burdened by the caregiving role. Other caregivers reported that family members could provide advice on how they should comfort the care recipient. For some caregivers, whose family members lived overseas, emotional support was received and updated information about the care recipient shared, through use of digital technology.

#### Support from friendship networks

Some caregivers reported receiving emotional and informational support from their friendship networks (Table 2). Some received emotional support from friends who had also experienced a family member being diagnosed with cancer and who understood the treatment process. Caregivers also reported receiving information on reputable cancer resources through their friendship networks. For one caregiver, their friendship network also became a source of emotional support for the care recipient.

### Barriers to accessing emotional support from social networks

However, a number of caregivers did not want to rely on their networks for emotional support and perceived reliance on networks as unhelpful. Four subthemes emerged related to barriers to accessing emotional support from social networks: (a) responsibility to protect others from burden; (b) reliance on oneself and stoicism; (c) avoiding discussions as a coping mechanism; and (d) cancer, illness and death as taboo topics. These subthemes are detailed below.

#### Responsibility to protect others from burden

Many participants were reluctant to reach out to friends and family for emotional support, speaking of their responsibility to protect others from their distress and not wanting to contribute to their own burdens (Table 2). Caregivers believed that talking about their own stress and strain was an unfair burden to shift onto family members and friends. Caregivers identified the importance of maintaining an outward appearance of being positive and happy to ensure that family members and friends were not concerned for the caregiver’s wellbeing. As a result, caregivers tried to appear happy and optimistic in front of others, even if they experienced distress.

Caregivers also reported responsibility for the emotional wellbeing of others around them. As such, they believed that talking about their own stress and strain would negatively impact the happiness of others. Some caregivers also believed that talking about their distress would bring sadness to others, which would further exacerbate negative feelings in the caregiver.

Cultural and generational factors were also identified as reasons for the caregivers’ belief; it was their duty to protect others from burden. For people from Chinese communities, there was a tradition of discussing only the positive aspects of one’s circumstances within their networks. Caregivers identified that non-disclosure was the inter-generational approach to managing burden and stress, adopted by older generations to protect younger generations: Although older generations would avoid discussing difficulties with younger generation to protect them from stress and strain, caregivers also reported that this had the opposite effect by ill-equipping them to manage stressors in their life.

Notably, several caregivers identified that it was the first time they had spoken about their caregiving experience with others. However, they also reflected that it was useful to talk about their caregiving experience with others.

#### Reliance on oneself and stoicism

Caregivers reported the importance of relying on oneself to manage the stress, strain and burden as a result of providing care. For some caregivers, the reluctance to talk to their friends about their situation was due to the belief that friends would not be able to help, thus the need to rely on oneself. Caregivers also acknowledged that talking to social support networks about their distress could be a useful source of emotional support: Some caregivers preferred to depend on themselves to manage the situation and did not want to draw attention from others. Intertwined with reliance on oneself appeared to be a sense of stoicism and the need to manage the burden on their own; the caregiving role was not the responsibility of others. Some caregivers appeared to feel strongly that caring was their duty as a family member.

#### Avoiding discussions as a coping mechanism

However, several caregivers reported not talking about their caregiving situation despite feeling distressed (Table 2). One caregiver described not discussing their situation with others because it made them “feel weak” when they wanted to “be strong” (C20, Table 2). Rather than talking with others to manage their distress, some caregivers would keep their feelings to themselves.

#### Cancer, death and illness as taboo topics

Caregivers reported avoidance of discussing topics such as cancer, illness and death within their social networks (Table 2). Although there was recognition by caregivers that conversations about cancer should not be taboo, for some of their family members, it was considered to bring bad luck, and thus, cancer was rarely discussed within the family. For some caregivers, avoiding challenging conversations about cancer and death also appeared to be a coping mechanism against distress. Avoidance of talking about death was also considered a cultural norm. One caregiver reported that news of a family member’s death was not directly given to them when they were living abroad. Avoidance of discussing illness and health conditions among family members was also reported. Caregivers spoke of needing to piece together information about a family member’s chronic health condition (e.g. transplant recipient) on their own, despite its significant impact on their health and wellbeing.

### Isolation and loss of connection following the cancer diagnosis

Caregivers from both CALD groups lamented the physical separation from their informal support systems, with most of their wider family located overseas, which created perceived isolation and loneliness. For one caregiver, perceived isolation may have been further exacerbated by the care recipient’s wish to not disclose the cancer diagnosis to their family or social networks; thus, this caregiver was not able to discuss the cancer even with immediate family members.

Some participants reported that they could not rely on the care recipient for emotional support as they may have done prior to diagnosis and reported a lack of social connection. Caregivers also reported no longer being able to share their innermost thoughts with the care recipient following the diagnosis, which included being unable to talk about their concerns about the cancer.

### Faith as a source of emotional support

Several caregivers from Arabic communities identified relying on their faith and religion as a source of emotional support when providing care to someone with cancer. For one caregiver, this was in part due to lack of time to focus on their own needs. For other caregivers, faith was an important source of support for them when managing and making sense of the diagnosis.

### Utility of support groups and caregiver advocates

Participants from both CALD groups discussed the utility of formal caregiver support groups, with mixed views. Some caregivers found the concept of support groups useful to better understand care recipient experiences and to get information on the cancer. However, a number of participants reported negative perceptions about joining support groups. Some caregivers identified cultural or language differences as a barrier to accessing support groups and preferred to seek support from their existing social networks, rather than seeking help from strangers. Some caregivers believed joining support groups would not be helpful because they perceived other caregivers could not relate to their situation, and information given would not be relevant to their circumstances. Other caregivers believed that participating in support groups would negatively impact their own emotional wellbeing. One caregiver reported actively avoiding support groups because they feared overt displays of emotion.

Several participants agreed that connecting with a same-language support group would be useful. One caregiver described her loneliness at not feeling proficient enough in English to talk to other caregivers and that she was unable to find other Chinese-speaking caregivers in her area. Other caregivers suggested that connecting with a same-language peer support person with similar caregiving experience would be beneficial.

### Universality of providing care

In examining how culture informed participant’s caregiving experience, it appeared that the care recipient’s survival was the primary focus for caregivers, rather than cultural considerations: “We’re talking about life and death…so people are not worrying about culture that much anymore. I think cultural safety is more a topic, let’s say, when it comes to pregnancy…but in like, cancer treatment, we’re dying, like come on” (C15, Chinese, female, caring for mother). As such, it appeared that caregivers felt that the caring experience was somewhat universal and not dictated by cultural norms or tradition: “when this happens…[it] doesn't matter which background you are from…it’s…how much you are willing to give, how much you are willing to sacrifice” (C17, Arabic, female, caring for daughter).

## Discussion

The present study aimed to investigate the role of social support in optimising wellbeing for caregivers of people with cancer from Chinese and Arabic communities. The results showed that some caregivers relied on their family and friendship networks for emotional support. However, a number of barriers to seeking support from networks were also identified, and these appeared to be influenced by cultural and generational factors.

Similar to existing studies [34, 35], caregivers identified family members and friends as a source of emotional support; they relied on them when they felt distress or struggled with the burden of providing care to someone with cancer. Family members and friends were identified as providing spontaneous comfort, advice and care to caregivers, as well as informational support. A recent study [35] found that among people with cancer and their caregivers, four in five identified their family and friends as key sources of emotional support; however, up to one-third of participants also reported receiving insufficient emotional support to meet their needs. In the current study, caregivers reported that their family networks lived overseas, which may have created barriers to seeking timely emotional support due to time differences and accessibility of communication.

Similar to Chinese people living with cancer in Canada [36], caregivers in the current study identified the importance of appearing happy and strong in the presence of family members and friends, and not disclosing their own distress, in an effort to not burden others. Stoicism is an important predictor of the extent to which both patients with a wide variety of health challenges, and caregivers, share information about physical pain [37] and emotional burden with others [38, 39] and thereby likely moderates the potential positive impact of social support. Caregivers in our study also preferred to rely on themselves and spoke of a perceived sense of stoicism when managing the caregiving role. Further, several caregivers disclosed that participating in the study was the first time they had spoken about their caregiving experiences with others; however, these individuals also noted that it was useful for them to speak about these experiences.

Ways of coping may be influenced by cultural and generational factors; older generations may act to protect younger generations from distressing or burdensome information and vice versa. Notably, Chinese and Arabic cultures are considered collectivist [40]. Such cultures focus on the importance of maintaining relationships and norms within broader social systems, prioritising the wellbeing of the groups and the needs of valued others, over one’s own needs [41, 42]. This contrasts with individualist cultures (e.g., Western societies) in which individuals prioritise and focus on their own goals and interests [43]. The finding that caregivers of Chinese and Arabic speaking cancer patients were reluctant to share their distress with family members and friends may reflect, at least in part, the imperative in collectivist cultures to “save face” and maintain group harmony [44]. Saving face has be likened to adapting an avoidant coping strategies (c.f., distracting oneself, or avoiding a problem through minimisation or avoidance) [45], and, while this strategy can eliminate the burden placed on others, it will not relieve caregiver’s own distress and the burden of providing care. In addition, emotional control is considered a sign of maturity in collectivist cultures; therefore, showing emotion could be viewed as distasteful or inappropriate and, thereby, create further distress [45].

It is unknown whether stoicism is a greater influence on support seeking among CALD caregivers than among the general population of caregivers, and our findings suggest the utility of examining the impact of stoicism on support seeking outcomes between these populations. Nonetheless, stoicism, and not verbalising negative feelings to others when experiencing hardships, has been associated in Chinese cultures with respect seeking [46]. Research with Asian populations in the USA has documented how family support can buffer the effects of stress and thereby impact depression [45]. Encouraging caregivers from CALD communities to share their experiences with family members and friends may be a culturally acceptable strategy for managing emotional strain as long as stoicism can be surmounted.

Although some caregivers identified the usefulness of talking about their experiences with their social networks to cope with the cancer diagnosis and caregiving role, others avoided discussions about cancer and acknowledged that cancer, death, and illness were taboo topics. Reasons for avoiding conversations about cancer have been attributed among Chinese people in the UK to beliefs about the interconnected nature of the body and mind, in which an individual’s psychological state influences their physical health and vice versa [47]. According to these beliefs, evoking negative emotions is likely to potentially lead to disease; thus, avoiding discussions about cancer is likely a coping mechanism to protect against the negative impacts of the disease [47]. In addition, research shows that in Chinese and Arabic cultures, cancer is considered a taboo subject, and not discussed due to fear of the disease, perceived stigma, and shame brought upon the family [48, 49].

Negative views about talking about cancer may be attenuated through use of alternative wording (e.g. growth or tumour), which capture similar information; however, it reduces the negative connotations associated with the word “cancer” [49]. Although some studies report death is a taboo topic in CALD communities [50], other literature suggests that individuals from CALD communities are comfortable talking about death and may use euphemisms in discussions [51, 52]. Our finding that individuals avoided talking about death is likely intertwined with avoidance as a coping mechanism and wanting to protect others from experiencing burden. Avoidance in talking about cancer, even when used as a coping mechanism, can also be a barrier to CALD caregivers seeking help. Ensuring that health providers are aware of culturally sensitive strategies to discuss cancer-related topics with individuals affected by cancer from CALD communities has the potential encourage them to access to supportive care services to meet their emotional and supportive care needs.

Similar to existing studies [53, 54], caregivers from Arabic communities in the current study reported a reliance on their faith and religion as a source of support when managing the disease and caregiving role. Indeed, managing illness is often considered a test from God [55]. The findings suggest the importance of including holistic strategies and consideration of religious beliefs and resources when providing support to people affected by cancer from Arabic communities.

Mixed opinions were reported about the potential utility of formal support groups for caregivers of people from CALD communities, with language and cultural differences identified as barriers to their access. Negative views of relevance of the information shared and how attendance at support groups might impact caregivers’ own wellbeing were also identified. However, same-language support groups or connecting with a same language peer-support persons were considered by some participants as potentially useful support. Further investigations into how, and whether, structured in-language formal peer-support programs are useful to caregivers from CALD communities is warranted.

### Limitations and future directions

A strength of this study is its qualitative approach with targeted representatives from two language groups. Semi-structured interviews captured the lived experiences of the target population, which may be missing in quantitative designs. Nonetheless, the study is exploratory, and findings cannot be considered generalisable to all caregivers from CALD communities. Specifically, the study included caregivers from Chinese (*n* = 12) and Arabic (*n* = 12) communities only, and thus findings cannot be extrapolated to those from broader CALD groups. Only one-quarter of the sample were men, which may limit understanding of experiences of male caregivers from CALD communities. In addition, demographic and carer characteristics were not collected from those who declined participation; thus, it is unclear whether there were differences in those who participated in the study compared with those who did not participate. Furthermore, the study may have been limited by the differences in language and background between the non-CALD interviewer and CALD participants. It is possible that interviews conducted in participants’ first language may have captured more nuance. Furthermore, it is not known if participants felt wholly comfortable sharing their stories with a non-CALD interviewer. Indeed, results showed that participants were more comfortable seeking *support* from a person with a similar background and language, and it is possible that a similar principle applies in the research setting.

Further research is needed to test how caregivers from CALD communities can optimally access and receive emotional support from social networks. Notably, despite barriers and reluctance to disclose their caregiving circumstances with others, participants also identified the usefulness of speaking about their experiences, suggesting that seeing emotional support from their social networks may be beneficial for their health and wellbeing. Participants in this study agreed that access to peer networks who speak their first language would be useful. Additional research is also needed to examine how barriers to seeking support from social networks can be addressed in caregivers from CALD communities, in particular because they appear cultural and generational. Given advancements in digital technology, investigating the feasibility and utility of online support platforms tailored to the needs of CALD caregivers to provide virtual support networks, alleviating loneliness is suggested. Further, understanding the relevance of and the development and testing of culturally relevant strategies to encourage caregivers from CALD communities to seek emotional support from professional avenues is also warranted. Further, additional research which compares experiences of caregivers from additional CALD communities has the potential to identify parallels and variations in their social support needs, as well as and the cultural challenges they confront. Such research has the potential to inform the development of community-specific initiatives to improve wellbeing in caregivers from CALD communities. Understanding the long-term effects of unmet social and emotional support on caregiver wellbeing and care recipient health outcomes is also recommended. Comparing and contrasting whether social support is used differently for emotional wellbeing between CALD and non-CALD caregivers is also warranted. Finally, additional research is needed to examine the experiences of caregivers that do not speak English, as the emotional support needs among these caregivers may differ.

## Conclusions

Caregivers identified a number of cultural and generational barriers to seeking emotional support from their social networks and were often isolated from their support system, finding it difficult to disclose their situation and seek emotional support. Cancer services must recognise that informal caregivers play a significant role in the treatment journey and that CALD caregivers have an additional burden in seeking informal support for themselves. Findings from the current study have the potential to inform the development of interventions, strategies, and resources that are tailored to cultural beliefs and address barriers that impact CALD caregivers in order to enhance their psychological wellbeing. Empirical testing of culturally appropriate strategies that improve social support seeking among caregivers from CALD communities is recommended.
